# Gene expression of the IGF pathway family distinguishes subsets of gastrointestinal stromal tumors wild type for KIT and PDGFRA

**DOI:** 10.1002/cam4.57

**Published:** 2013-02-03

**Authors:** Carol Beadling, Janice Patterson, Emily Justusson, Dylan Nelson, Maria A. Pantaleo, Jason L. Hornick, Matias Chacón, Christopher L. Corless, Michael C. Heinrich

**Affiliations:** 1 Knight Cancer Institute, Oregon Health and Science UniversityPortland, Oregon; 2 Division of Hematology and Oncology, Oregon Health and Science UniversityPortland, Oregon; 3 Department of Hematology and Oncology Sciences, S. Orsola-Malpighi Hospital, University of BolognaItaly; 4 Department of Pathology, Brigham and Women's Hospital, Harvard Medical SchoolBoston, Massachusetts; 5 GATE-D, GrupoArgentino de Tumores Estromales DigestivosBuenos Aires, Argentina; 6 Department of Pathology, Oregon Health and Science UniversityPortland, Oregon; 7 Portland VA Medical CenterPortland, Oregon

**Keywords:** Gastrointestinal stromal tumor, IGF1R, wild type

## Abstract

Gastrointestinal stromal tumors (GISTs) arise from the interstitial cells of Cajal (ICCs) and are the most common mesenchymal neoplasm of the gastrointestinal tract. While the majority of GISTs harbor activating mutations in either the v-kit Hardy-Zuckerman feline sarcoma viral oncogene homolog (KIT) or platelet-derived growth factor receptor alpha (PDGFRA) tyrosine kinases, approximately 10–15% of adult GISTs and 85% of pediatric GISTs lack such mutations. These “wild-type” GISTs have been reported to express high levels of the insulin-like growth factor 1 receptor (IGF1R), and IGF1R-targeted therapy of wild-type GISTs is being evaluated in clinical trials. However, it is not clear that all wild-type GISTs express IGF1R, because studies to date have predominantly focused on a particular subtype of gastric wild-type GIST that is deficient in the mitochondrial succinate dehydrogenase (SDH) complex. This study of a series of 136 GISTs, including 72 wild-type specimens, was therefore undertaken to further characterize wild-type GIST subtypes based on the relative expression of transcripts encoding IGF1R. Additional transcripts relevant to GIST biology were also evaluated, including members of the IGF-signaling pathway (IGF1, IGF2, and insulin receptor [INSR]), neural markers (CDH2[CDH: Cadherin], neurofilament, light polypeptide, LHX2 [LHX: LIM homeobox], and KIRREL3 [KIRREL: kin of IRRE like]), KIT, PDGFRA, CD34, and HIF1A. Succinate dehydrogenase complex, subunit B protein expression was also assessed as a measure of SDH complex integrity. In addition to the previously described SDH-deficient, IGF1R^high^ wild-type GISTs, other SDH-intact wild-type subpopulations were defined by high relative expression of IGF1R, neural markers, IGF1 and INSR, or low IGF1R coupled with high IGF2. These results underscore the complexity and heterogeneity of wild-type GISTs that will need to be factored into molecularly-targeted therapeutic strategies.

## Introduction

The identification of activating mutations in the v-kit Hardy-Zuckerman feline sarcoma viral oncogene homolog (KIT) or platelet-derived growth factor receptor alpha (PDGFRA) tyrosine kinases in ~85% of gastrointestinal stromal tumors (GISTs) enabled the introduction of targeted therapies that have proven much more effective than traditional chemotherapy in the treatment of advanced disease (reviewed in [1]). However, approximately 10–15% of adult GISTs and the majority of pediatric GISTs lack KIT or PDGFRA mutations [2,3]. These so-called wild-type GISTs tend to respond less favorably to kinase inhibitors than kinase-mutated GISTs [4–6]. This is significant because up to 40% of GIST patients will suffer disease recurrence after primary tumor resection [7]. A more comprehensive characterization of the molecular GIST subtypes is therefore needed to advance clinical management of GIST.

Approximately one-fourth of adult wild-type GISTs and the majority of pediatric GISTs comprise a common subtype of wild-type GIST. These occur most frequently in females, arise in the stomach, are multinodular, and exhibit epithelioid or mixed epithelioid and spindle-type morphology [8]. The prognosis in these cases is not predicted by the standard criteria of size or mitotic rate, and despite frequent lymph node metastases, these tumors run an indolent course. These GISTs do not respond well to imatinib, so that alternate therapeutic strategies are needed [8,9].

At the molecular level, this wild-type GIST subpopulation is characterized by loss of expression of the mitochondrial succinate dehydrogenase (SDH) complex and high expression of the insulin-like growth factor 1 receptor (IGF1R). Mutational inactivation or loss of any SDH component (A, B, C, or D) results in loss of the succinate dehydrogenase complex, subunit B (SDHB) subunit, so that SDHB immunohistochemistry may be used as a surrogate to identify these GISTs [10–20]. The SDH complex converts succinate to fumarate. The succinate that accumulates when SDH complex activity is impaired leads to reduced turnover of hypoxia-induced factor 1 alpha (HIF1Α) and heightened expression of HIF1A target genes including vascular endothelial growth factor [21]. The IGF1R tyrosine kinase is activated when the receptor binds the ligands IGF1 or IGF2, triggering enhanced cell proliferation and survival through downstream activation of mitogen-activated protein kinase and PI3K-signaling pathways [22]. IGF1R is highly expressed at both the RNA and protein level in wild-type GISTs, and the receptor is activated, although not mutated in these tumors [23–29]. SDHB-deficient, IGF1R^high^ GISTs may therefore be considered candidates for therapies targeting VEGFR and/or IGF1R.

SDH-deficient, IGF1R^high^ wild-type GISTs may derive from a common progenitor, and the cell of origin may affect responses to targeted therapies. GISTs are hypothesized to originate from either myenteric (ICC-MY) or intramuscular (ICC-IM) interstitial cells of Cajal (ICCs) [30]. In the mouse, early ICC progenitors have a KIT^low^CD44^+^CD34^+^INSR^+^IGF1R^+^ phenotype, while committed progenitors are KIT^high^CD44^+^CD34^+^INSR^+^IGF1R^+^, and mature ICCs are KIT^high^CD44^+^CD34^−^INSR^−^IGF1R^−^ [31,32]. Unlike mature ICCs, the KIT^low^CD44^+^CD34^+^ stem cells are resistant to KIT inhibition, even in the presence of an activating KIT mutation.^31^ The high IGF1R expression in the ICC progenitors suggests that these may be the cells of origin of wild-type SDH-deficient, IGF1R^high^ GISTs. Indeed, SDH-deficient wild-type GISTs exhibit a gene expression pattern more closely related to immature than mature ICCs, including high relative levels of IGF1R as well as several genes associated with a neural phenotype [33].

While the molecular characterization of SDH-deficient, IGF1R^high^ wild-type GISTs has pointed the way toward potential novel therapeutic strategies for this population, it is not clear that these strategies would be applicable broadly to all wild-type GISTs. In the case of IGF1R-targeted therapy, for example, it has not been established that all wild-type GISTs express IGF1R, nor that all IGF1R^high^ GISTs are SDH deficient. Heterogeneity among wild-type GISTs could impact on their response to IGF1R-targeted therapy.

Therefore, the goal of this study was to determine whether wild-type GIST populations other than the well-characterized SDH-deficient IGF1R^high^ type could be defined by examining the expression of genes relevant to GIST biology. The relative expression of transcripts encoding IFG1R, additional members of the IGF-signaling pathway, neural markers, KIT, PDGFRA, CD34, and HIF1A was evaluated in a series of 72 wild-type tumors. An additional group of 64 KIT- or PDGFRA-mutant cases was also included as a comparator. SDHB protein expression was evaluated in 81 of these cases. In addition to the wild-type SDH-deficient, IGF1R^high^ population, distinct SDH-intact wild-type GIST subpopulations were identified with high relative expression of IGF1R, neural markers, IGF1 and insulin receptor (INSR), or low IGF1R combined with high IGF2. These results demonstrate that there are several wild-type GIST subtypes that may need to be considered in the optimization of therapeutic strategies.

## Materials and Methods

### Tumor specimens and cDNA preparation

This study was conducted under full Institutional Review Board (IRB) approval per federal and institutional guidelines. Blocks of formalin-fixed, paraffin-embedded (FFPE) tumor tissue, or unstained sections of FFPE tissue, were obtained from the Pathology archives of the Oregon Health and Science University, the Grupo Argentino de Tumores Estromales Digestivos, Buenos Aires, Argentina, and the Clinical Centre of Serbia, Belgrade. Wild-type cases were selected from several population-based or therapeutic intervention studies for which we received samples over the past 10 years. All wild-type cases with sufficient residual FFPE specimens for RNA extraction were included in this study. Additional KIT- or PDGFRA-mutant cases from these series were included to form a comparison group for the wild-type GIST cases. KIT (exons 9, 11, 13, and 17) and PDGFRA (exons 12, 14, and 18) genotypes were previously determined as described [2,34]. Tumor-rich areas (≥90%) were scraped from 5-μm unstained sections by comparison with a hematoxylin and eosin–stained slide, and paraffin was removed by incubation in Q solution (TrimGen, Sparks, MD). Total RNA was extracted using a High Pure FFPE RNA Micro kit (Roche, Indianapolis, IN). Single-stranded cDNA was prepared from 1 μg of total RNA in a 50-μL reaction using 60 μmol/L random hexamer primers, 0.5 mmol/L dNTPs, 100 units RNaseOUT, 5 mmol/L DTT (DTT: dithiothreitol), 1× First Strand buffer, and 500 units SuperScript III reverse transcriptase following manufacturer's instructions (Invitrogen by Life Technologies, Grand Island, NY).

### Real-time polymerase chain reaction

Real-time polymerase chain reaction (PCR) was carried out in a 20-μL reaction using 2-μL single-stranded cDNA (corresponding to 40 ng initial total RNA) and 1× Probes Master MIX (Roche), with a FAM-labeled hydrolysis probe specific to the target of interest, and a Texas Red-labeled probe specific to the reference Glyceraldehyde 3-phosphate dehydrogenase (GAPDH). Cycling conditions on a LightCycler 480 instrument (Roche) included 10 min at 95°C followed by 40 cycles of 95°C for 10 sec and 60°C for 20 sec. Custom primers and hydrolysis probe were used to detect a 66-bp GAPDH amplicon: GAPDH forward CACTAGGCGCTCACTGTTCT, GAPDH reverse GCGAACTCCCCGTTG, GAPDH probe 5′TexRd-XN/TGGGGAAGGTGAAGGTCGGA/3′IAbRQSp (IDT, Coralville, IA). Additional targets were detected using commercial TaqMan Gene Expression assays (Applied Biosystems by Life Technologies): IGF1R (64-bp amplicon) assay Hs00609566_m1, IGF1 (63 bp) Hs01547657_m1, IGF2 (81 bp) Hs01005963_m1, INSR (76 bp) Hs00965956_m1, IRS1 (insulin receptor substrate [IRS]) (69 bp) Hs00178563_m1, IRS2 (70 bp) Hs00275843_s1, CDH2 [Cadherin (CDH)] (78 bp) Hs00983062_m1, neurofilament, light polypeptide (NEFL) (71 bp) Hs00196245_m1, LHX2 (LIM homeobox [LHX]) (64 bp) Hs01104717_m1, KIRREL3 kin of IRRE like (KIRREL) (68 bp) Hs01123170_m1, CD34 (61 bp) Hs00990730_m1, and HIF1A (62 bp) Hs00936371_m1.

Plasmid controls were used to confirm that the assays were linear over a series of template dilutions from 3 to 3 × 10^6^ copies (linear regression *R*^2^ ≥0.98 for all assays). Plasmids were prepared by cloning the PCR assay amplicons or were obtained commercially. Commercial plasmids (OriGene, Rockville, MD) included clones of IGF1 (RG215257), IGF2 (RC201849), INSR (RG215257), IRS1 (SC124032), CDH2 (SC119018), NEFL (SC116280), LHX2 (SC126761), KIRREL3 (SC100705), CD34 (SC302353), and HIF1A (SC119189). Plasmid standards were also included in parallel with each specimen test. The plasmid standards were used to calculate the relative expression of each target as the ratio of copies of the target of interest compared with copies of GAPDH.

Cluster analysis of relative gene expression profiles was performed with Cluster 3.0, and results were visualized with TreeView software [35]. Expression values were log_2_-transformed and normalized, and cluster analysis was performed with Self-Organizing Maps and a Euclidean distance measure. A *t*-test was used to compare relative gene expression levels between specimen groups across all genes (Table S2).

### SDHB immunohistochemistry

Sufficient tissue was available from 81 specimens to evaluate SDHB protein. SDHB protein expression was measured by immunohistochemistry with a monoclonal mouse anti-SDHB antibody (1:100 dilution, clone 21A11AE7, Abcam, Cambridge, MA) on 4-μm-thick FFPE whole-tissue sections following pressure cooker antigen retrieval (0.001 mol/L citrate buffer, pH 6.0), as described previously [15].

## Results

### IGF1R expression in molecularly and clinically defined GIST subtypes

**Figure 1:**
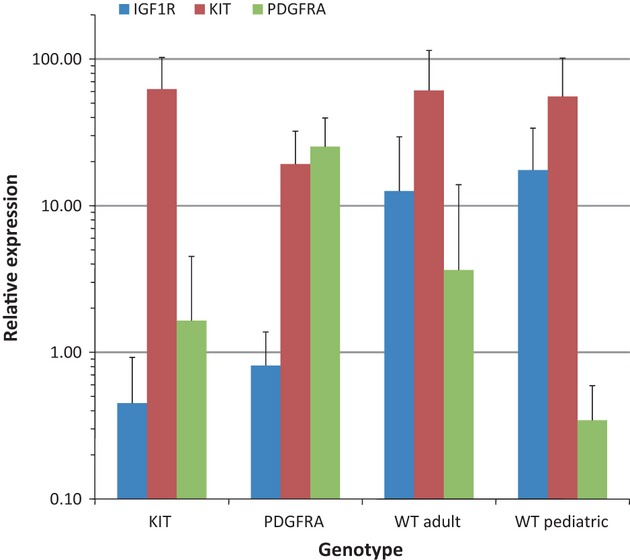
Relative KIT, PDGFRA, and IGF1R expression in KIT-mutant, PDGFRA-mutant, and wild-type GISTs. Target gene expression was measured in 138 GIST specimens by RT-PCR relative to the reference GAPDH. Values are expressed as the percent ratio relative to GAPDH, and depict the mean ± standard deviation for each GIST subtype. PDGFRA, platelet-derived growth factor receptor alpha; IGF1R, insulin-like growth factor 1 receptor; GIST, gastrointestinal stromal tumor; RT-PCR, real-time polymerase chain reaction.

To evaluate gene expression patterns in GIST subtypes, multiplex RT-PCR assays were used to measure the expression of a series of target genes relative to the reference gene GAPDH. The assays employed a FAM-labeled probe to detect the target of interest, and a Texas Red-labeled probe to measure GAPDH. To validate the approach, we first measured relative KIT and PDGFRA expression, expressed as the percent ratio relative to GAPDH. A series of 136 GISTs was evaluated that included 52 KIT-mutant, 12 PDGFRA-mutant, 65 wild-type GISTs arising in adults, and seven wild-type GISTs arising in pediatric patients (Fig. 1). The relative KIT expression was approximately threefold lower in PDGFRA-mutant compared with KIT-mutant and wild-type GISTs (PDGFRA mutant vs. KIT mutant *P* = 5 × 10^−4^, vs. adult wild type *P* = 9 × 10^−3^, vs. pediatric wild type *P* = 2 × 10^−2^), while relative PDGFRA expression was approximately 7- to 70-fold higher in PDGFRA-mutant GISTs compared with the other subtypes (PDGFRA mutant vs. KIT mutant *P* = 2 × 10^−16^, vs. adult wild type *P* = 2 × 10^−8^, vs. pediatric wild type *P* = 3 × 10^−4^). These observations are consistent with previous reports of gene expression profiling using microarray methodology [36], and provide validation of the RT-PCR assays.

To determine the influence of kinase genotype on IGF1R expression in GIST, we used a similar RT-PCR assay to measure IGF1R relative to GAPDH. Measurement of IGF1R mRNA by RT-PCR has been shown to correlate with protein detection by immunohistochemistry [37]. As shown in Figure 1, the mean relative IGF1R expression was approximately 15- to 40-fold higher in wild-type specimens compared with KIT- or PDGFRA-mutant specimens, with highest expression in the pediatric wild-type cases. The mean values in adult wild-type (12.6 ± 16.9) and pediatric wild-type GISTs (17.5 ± 16.3) were both significantly higher than the values in KIT- (0. 5 ± 0.5, *P* = 1 × 10^−6^ vs. adult wild type; *P* = 7 × 10^−11^ vs. pediatric wild type) and PDGFRA-mutant specimens (1.6 ± 2.9; *P* = 2 × 10^−2^ vs. adult wild type; *P* = 2 × 10^−3^ vs. pediatric wild type). These results are consistent with previous reports of high relative IGF1R expression in adult and pediatric wild-type GISTs [23, 26, 28, 29].

### Expression patterns of members of the IGF-signaling pathway, neural markers, CD34, and HIF1A further define distinct GIST subtypes

**Figure 2:**
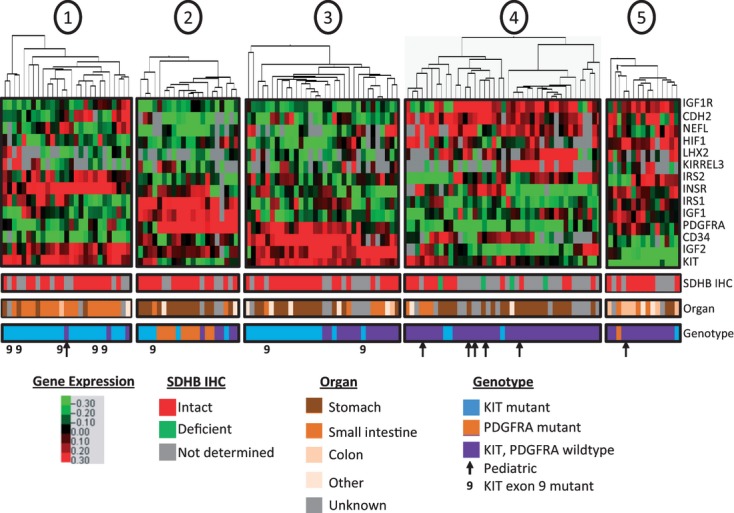
Gene expression clusters define GIST subtypes. Self-organizing cluster analysis of the relative expression of 14 target genes relative to GAPDH identified five expression groups among 138 GIST specimens. Red indicates high relative expression and green indicates low relative expression. Location of the primary tumor is listed as stomach, small intestine, other, or unknown. SDHB immunohistochemistry was performed on 82 of the specimens, and was categorized as intact or deficient, as indicated. KIT and PDGFRA mutation status are illustrated in blue or orange, respectively, with nine indicating KIT exon 9 mutations. Pediatric specimens are indicated by arrows. GIST, gastrointestinal stromal tumor; PDGFRA, platelet-derived growth factor receptor alpha.

To further define the GIST subtypes, the expression of 11 other genes in addition to KIT, PDGFRA, and IGF1R was also measured relative to GAPDH. The additional targets included members of the IGF-signaling pathway (IGF1, IGF2, INSR, IRS1, IRS2), neural markers (CDH2, LHX2, NEFL, KIRREL3), as well as CD34 and HIF1A. Self-organizing cluster analysis revealed five cluster groups defined by the expression patterns of the 14 genes in the 136 GISTs (Fig. 2, Tables 1 and S2). The majority of wild-type GISTs segregated into three clusters, two of which exhibited high relative IGF1R expression and a third which exhibited low IGF1R expression that is comparable to KIT- and PDGFRA-mutant GISTs. The KIT-mutant GISTs clustered in two main groups, one of which was shared with wild-type GISTs. These two KIT-mutant groups were distinguished from each other by differences in CD34 expression. The majority of PDGFRA-mutant GISTs clustered in one group defined by high relative PDGFRA, IGF1, and IRS1 expression.

**Table 1:** Target gene expression relative to GAPDH in GIST subtypes

	Heatmap cluster (genotype)					
Gene	1 (KIT)	2 (PDGFRA)	3 (KIT)	3 (WT)	4 (WT)	5 (WT)
IGF1R	0.54 ± 0.56	0.79 ± 0.59	0.26 ± 0.16	0.48 ± 0.48	20 ± 18	13 ± 16
IGF1	0.42 ± 0.51	8.2 ± 5.5	0.48 ± 0.67	0.41 ± 0.36	1.8 ± 1.7	3.1 ± 5.8
IGF2	235 ± 267	568 ± 613	334 ± 414	1680 ± 1187	402 ± 821	34 ± 85
INSR	0.49 ± 0.33	0.69 ± 0.98	0.15 ± 0.07	0.12 ± 0.12	0.28 ± 0.28	0.55 ± 0.60
IRS1	1.2 ± 2.3	12 ± 10	0.73 ± 1.0	1.1 ± 1.5	1.2 ± 2.4	1.8 ± 2.3
IRS2	7.0 ± 8.1	7.3 ± 13.2	6.4 ± 17.4	3.3 ± 3.2	17 ± 27	39 ± 72
CDH2	0.53 ± 0.74	0.050 ± 0.053	0.89 ± 2.1	0.82 ± 1.3	5.8 ± 4.3	11 ± 17
LHX2	0.073 ± 0.180	0.039 ± 0.060	0.085 ± 0.229	0.12 ± 0.18	5.2 ± 8.3	1.3 ± 3.7
NEFL	0.059 ± 0.167	0.023 ± 0.037	0.028 ± 0.078	1.6 ± 4.2	8.0 ± 13.7	2.8 ± 6.9
KIRREL3	0.031 ± 0.079	0.036 ± 0.037	0.007 ± 0.009	0.061 ± 0.122	1.9 ± 4.2	0.15 ± 0.36
KIT	53 ± 35	21 ± 13	62 ± 32	70 ± 36	83 ± 54	2.4 ± 3.4
PDGFRA	0.28 ± 0.35	26 ± 15	2.5 ± 3.2	3.4 ± 6.3	0.76 ± 1.5	1.5 ± 1.5
CD34	4.6 ± 4.2	24 ± 15	73 ± 39	84 ± 61	62 ± 69	10 ± 13
HIF1	3.5 ± 2.1	4.2 ± 1.2	2.4 ± 0.9	4.4 ± 2.3	9.5 ± 5.5	12.9 ± 6.9

Values are expressed as the percent ratio of the target gene relative to GAPDH. Numbers are the mean ± standard deviation. GIST, gastrointestinal stromal tumor; PDGFRA, platelet-derived growth factor receptor alpha; WT, wild type; IGF1R, insulin-like growth factor 1 receptor; HIF1, hypoxia-induced factor 1.

The groups defined by gene expression patterns were in some cases paralleled by the location of the primary tumors. For example, among 24 KIT-mutant tumors in gene expression group 1, 16 originated in the small intestine, while 12 of the 18 KIT-mutant tumors in gene expression group 3 were from the stomach (Table S1). High relative CD34 expression in the gastric GISTs was one of the discriminators between the two KIT-mutant groups, consistent with reports of high CD34 expression in GISTs of the stomach versus small intestine [38,39]. Among the adult wild-type GISTs, 19 tumors were located in the stomach. Fifteen of these 19 specimens were clustered in gene expression group 4, characterized by high relative expression of IGF1R, neural markers, IGF1, and INSR. The correlation of tumor location with gene expression profiles suggests that these gene expression patterns may define biologically distinct GIST subtypes.

### Wild-type GIST subtypes

The wild-type GISTs were predominantly clustered in three groups that encompassed 65 of the 72 total wild-type specimens. These included 59 adult and six pediatric wild-type specimens (Fig. 2). Cluster groups 4 and 5 included 51 of the wild-type specimens and exhibited high average relative IGF1R expression, while the average relative IGF1R expression was approximately 25- to 40-fold lower in the 14 wild-type specimens of group 3 (group 3 vs. group 4: *P* = 3 × 10^−4^, group 3 vs. group 5: *P* = 6 × 10^−3^). IGF1R expression in the IGF1R^low^ wild-type group was comparable with that observed in KIT- and PDGFRA-mutant cases (Table 1). Thus, approximately one fifth of the wild-type GISTs do not express elevated levels of IGF1R.

The two wild-type GIST groups with high relative IGF1R expression could be further discriminated from the wild-type IGF1R^low^ group by the elevated relative expression of neural markers among the IGFIR^high^ GISTs (Table 1). Mean relative CDH2 expression among the wild-type GISTs in cluster group 4 was 6- and 100-fold greater than in the KIT- or PDGFRA-mutant GISTs, respectively (*P* < 7 × 10^−5^), and was similarly elevated in the wild-type GISTs of cluster group 5 (*P* < 2 × 10^−2^ vs. KIT mutant, *P* = 5 × 10^−2^ vs. PDGFRA mutant). In contrast, the mean relative CDH2 expression in the IGF1R^low^ GISTS of group 3 was comparable with that of the KIT- and PDGFRA-mutant cases, and was approximately 10-fold lower than in the IGF1R^high^ wild-type GISTs of group 4 (*P* = 1 × 10^−4^) or group 5 (*P* = 4 × 10^−2^). Similarly, LHX2 mean relative expression was approximately 15- to 130-fold higher in the wild-type GISTs of cluster groups 4 and 5 compared with KIT- or PDGFRA-mutant GISTs, or the wild-type IGF1R^low^ GISTS of group 3, although this difference only reached statistical significance in comparison with the wild-type GISTs of cluster group 4 (*P* < 5 × 10^−2^). KIRREL3 mean relative expression was also higher in the wild-type IGF1R^high^ GISTs than in the wild-type IGF1R^low^ or KIT- and PDGFRA-mutant GISTs, although the difference only reached statistical significance in the comparison of cluster group 4 and KIT-mutant GISTs (*P* = 4 × 10^−2^). Finally, NEFL expression was highest in the wild-type GISTs of cluster group 4, intermediate in the wild-type GISTs of cluster groups 5 and 3, and lowest in the KIT- and PDGFRA-mutant GISTs. These differences only reached statistical significance between the wild-type GISTs of cluster group 4 and the KIT-mutant GISTs (*P* < 3 × 10^−2^). Thus, in the case of all four neural markers, there was either a trend toward higher expression or a statistically significant elevation of mean relative expression in the IGF1R^high^ GISTs compared with the IGF1R^low^ or KIT- and PDGFRA-mutant GISTs.

Relative expression of members of the IGF-signaling pathway further distinguished the wild-type GIST subtypes (Table 1). The mean relative IGF1 expression was approximately four- to eightfold higher in the IGF1R^high^ wild-type GISTS of cluster groups 4 and 5 compared with the IGF1R^low^ wild-type GISTs of cluster group 3 (group 4 vs. group 3: *P* = 5 × 10^−3^, group 5 vs. group 3: *P* = 9 × 10^−2^). Similarly, INSR mean relative expression was approximately two- to fivefold higher in the IGF1R^high^ wild-type GISTs compared with the IGF1R^low^ wild-type GISTs (group 4 vs. group 3: *P* = 6 × 10^−2^, group 5 vs. group 3: *P* = 8 × 10^−3^). In contrast, IGF2, which was highly expressed in all cases, exhibited approximately 50-fold higher expression in the IGF1R^low^ GISTs compared with the IGF1R^high^ GISTs of group 5 (*P* = 4 × 10^−5^) and approximately fourfold higher than the IGF1R^high^ GISTs of group 4 (*P* = 7 × 10^−5^). While mean relative IRS2 expression was approximately 5- to 10-fold higher in the IGF1R^high^ GISTs than the IGF1R^low^ GISTs, the range of expression was broad, and the differences did not reach statistical significance. IRS1 expression was not different among the wild-type GIST groups. Hence, the wild-type GISTs with high IGF1R expression exhibited higher IGF1 and INSR levels, while the wild-type IGF1R^low^ GISTs expressed higher levels of IGF2.

HIF1A mean relative expression was approximately two- to threefold higher in the IGF1R^high^ wild-type GISTs of cluster groups 4 and 5 compared with the wild-type IGF1R^low^ cluster 3 (*P* < 1 × 10^−2^) (Table 1). The KIT- and PDGFRA-mutant GISTs were similar to the IGF1R^low^ wild-type GISTs, and expressed approximately two- to fivefold lower relative HIF1A levels compared with the IGF1R^high^ wild-type GISTs (*P* < 6 × 10^−3^).

While the expression of neural marker, IGF-signaling pathway, and HIF1A genes was similar among the two clusters of IGF1R^high^ wild-type GISTs, the two groups differed in the expression of KIT and CD34 (Table 1). Mean relative KIT expression was approximately 35-fold higher in the wild-type GISTs of group 4 compared with group 5 (*P* = 3 × 10^−6^), and CD34 mean expression was approximately sixfold higher in group 4 compared with group 5 (*P* = 1 × 10^−2^). KIT and CD34 relative expression were comparable among the IGF1R^low^ wild-type GISTs of group 3 and the wild-type GISTs of group 4. Thus, relatively high KIT and CD34 expression distinguished the IGF1R^high^ group 4 wild-type GISTs from the IGF1R^high^ group 5, and this high KIT and CD34 expression was shared with the wild-type IGF1R^low^ group 3 GISTs.

### SDHB protein expression

Sufficient tissue was available to measure SDHB protein levels by immunohistochemistry in 81 of the 136 GISTs, including 30 adult wild-type and one pediatric wild-type specimen. Only four specimens were deemed SDHB deficient, and all four were wild-type tumors with gene expression patterns that clustered in gene expression group 4 (Fig. 2). Three of the SDHB-deficient tumors were located in the stomach, two arising in adults and one in a pediatric patient. The fourth was a tumor located in the omentum of an adult. The average age of these four patients at the time of diagnosis was 33 years (range 18–47). In comparison, there were 11 wild-type GISTs in gene expression group 4 that exhibited intact SDHB expression. Five of these tumors were located in the stomach, three in the small intestine, one in the duodenum, and two of unknown location. The age at diagnosis was known for eight of these patients, and the average was 44 years (range 17–66). The difference in average age of patients with SDHB-deficient or SDHB-intact tumors was not significant (*P* = 0.2).

Of the 14 genes analyzed, only IGF2 relative expression exhibited a difference between SDHB-deficient and SDHB-intact wild-type specimens that reached statistical significance. The mean relative IGF2 level was 50 ± 11 in the four SDHB-deficient specimens (range 38–59) and 160 ± 113 in the 11 SDHB-intact wild-type specimens (range 38–318; *P* = 0.04). In comparison, the mean relative IGF1R expression was high in both the SDHB-deficient wild-type GISTs (mean 11 ± 9, range 4–23) and the SDHB-intact specimens (mean 16 ± 17, range 0.05–50, *P* = 0.5). There was also no significant difference in relative HIF1A expression between the SDHB-deficient (mean 7 ± 4, range 3–13) and SDHB-intact (mean 9 ± 5, range 3–23) wild-type GISTs of expression group 4 (*P* = 0.2). Thus, although the sample size was small, SDHB-deficient GISTs exhibited an approximately threefold lower relative IGF2 expression level compared with SDHB-intact wild-type GISTs. The SDHB-deficient tumors expressed high relative levels of IGF1R, but not all IGF1R^high^ wild-type GISTs were SDHB deficient.

### KIT-mutant GIST subtypes

Forty-two of the KIT-mutant specimens clustered in two groups, with 24 in group 1 and 18 in group 3. The latter group was shared with 14 of the IGF1R^low^ wild-type GISTs (Fig. 2, Table 1). Mean relative CD34 expression was approximately 16-fold higher in the KIT-mutant GISTs of group 3 compared with the KIT-mutant GISTs of group 1 (*P* = 3 × 10^−10^). The wild-type GISTs of group 3 had similar levels of CD34 as the KIT-mutant specimens of group 3, also 16-fold greater than that in the KIT-mutant GISTs of group 1 (*P* = 3 × 10^−7^). Similarly, mean relative PDGFRA expression was approximately 10-fold higher in the KIT-mutant or wild-type GISTs of group 3 compared with the KIT-mutant GISTs of group 1 (*P* < 2 × 10^−2^). In contrast, mean relative INSR expression was approximately three- to fourfold higher in the KIT-mutant GISTs of group 1 compared with either the KIT-mutant or wild-type GISTs of group 3 (both *P* < 3 × 10^−4^). In addition, five of eight KIT exon 9-mutant specimens segregated in expression group 1, along with the majority of KIT-mutant specimens localized in the small intestine. Thus, the KIT-mutant GISTs of groups 1 and 3 could be discriminated by higher relative expression of CD34 and PDGFRA in group 3, and higher relative INSR expression in group 1. The patterns of CD34, PDGFRA, and INSR expression were shared between the wild-type and KIT-mutant GISTs of group 3. The majority of KIT-mutant GISTs in group 1 were located in the small intestine, while those in group 3 were predominantly gastric.

### PDGFRA-mutant GISTs

Of the 12 PDGFRA-mutant GISTs, 11 clustered in expression group 2 (Fig. 2, Table 1). These specimens were characterized by mean relative PDGFRA expression that was approximately 10- to 100-fold greater than that of KIT-mutant or wild-type GISTs (*P* ≤ 3 × 10^−5^). The PDGFRA-mutant GISTs also expressed mean relative levels of IGF1 and IRS1 that were 5- to 20-fold higher than that expressed in KIT-mutant or wild-type GISTs. In the case of IGF1, these differences reached statistical significance in all comparisons of PDGFRA-mutant specimens (*P* = 9 × 10^−8^ to 4 × 10^−2^). Differences in IRS1 expression between PDGFRA mutant and all other groups also reached statistical significance (*P* = 3 × 10^−8^ to 8 × 10^−4^). Hence, PDGFRA-mutant GISTs were characterized by high relative expression of PDGFRA, IGF1, and IRS1.

## Discussion

**Figure 3:**
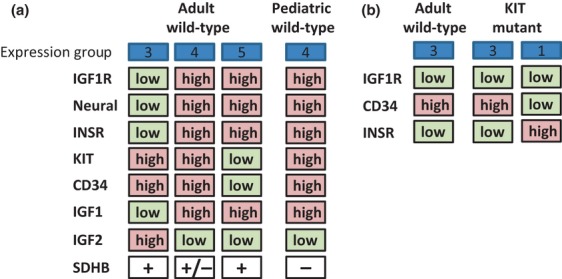
Subpopulations of wild-type and KIT-mutant GISTs. (a) Adult wild-type GISTs are segregated into four groups based on the expression levels of the IGF1R, neural marker, INSR, KIT, CD34 and IGF2 genes, and SDHB protein. Pediatric GISTs most closely resemble the adult wild-type GISTs with high relative expression of IGF1R, neural markers, INSR, KIT, and CD34, and low relative expression of IGF2. SDHB protein was detected in 16/19 IGF1R^high^ adult wild-type GISTs. (b) Some KIT-mutant GISTs exhibit similarities in relative CD34 and INSR expression compared with adult wild-type IGF1R^low^ GISTs. GIST, gastrointestinal stromal tumor; IGF1R, insulin-like growth factor 1 receptor.

Hierarchical cluster analysis of the relative expression of 14 genes relevant to GIST, including IGF1R, additional IGF-signaling pathway genes, neural markers, HIF1A, KIT, PDGFRA, and CD34, in 136 GISTs identified five distinct expression groups. Wild-type GISTs were stratified into three gene expression groups, including two with high relative IGF1R expression. SDHB protein expression was deficient in a subset of the IGF1R^high^ wild-type GISTs (Fig. 3a). In comparison, the KIT-mutant GISTs were separated into two groups, one of which was shared with IGF1R^low^ wild-type GISTs (Fig. 3b), while PDGFRA-mutant GISTs were categorized in a single group characterized by high relative expression of PDGFRA, IGF1, and IRS1.

The 38 wild-type specimens within gene expression group 4 exhibited high relative expression of IGF1R, neural markers, INSR, KIT, CD34, and IGF1, and low relative IGF2. Four of these tumors were SDHB-deficient GISTs, which have been well characterized as gastric tumors with multinodular epithelioid morphology and an indolent clinical course [14,15]. Eleven of the wild-type specimens in this group 4 were SDHB intact (the others were not tested), and included tumors in the stomach, small intestine, and duodenum. Two other subtypes of wild-type GIST were described by gene expression groups 3 and 5. The former exhibited high relative mean expression of KIT, CD34, and IGF2, with low relative IGF1R, INSR, IGF1, and neural markers. This group likely corresponds to a recently described population of adult wild-type GISTs with high relative IGF2 expression and low IGF1R expression [25]. Of note, high IGF2 is a poor prognostic indicator for GIST [40]. The wild-type specimens in expression group 5 showed a reciprocal pattern of low relative mean expression of KIT, CD34, and IGF2, and high relative expression of IGF1R, INSR, IGF1, and neural markers. All 15 wild-type GISTs in groups 3 and 5 that were tested for SDHB protein expression showed intact SDHB.

In the case of SDHB protein deficiency, it has been proposed that mitochondrial dysfunction may trigger elevated IGF1R expression [41], potentially through a calcium- and calcineurin-dependent mechanism [42]. This hypothesis is consistent with the high level of IGF1R expression in pediatric wild-type GIST, and the high prevalence of SDHB deficiency in these tumors [13, 16, 23, 24, 26, 28, 29, 43, 44]. However, in the present series of 31 adult wild-type GISTs screened for SDHB protein, 16 exhibited high relative IGF1R expression, but only three of these were SDHB deficient. Among these 16, six exhibited relative IGF1R expression levels equal to or greater than the pediatric wild-type GISTs, and all six of these possessed intact SDHB protein. However, this does not preclude the possibility that mitochondrial dysfunction caused by a mechanism other than SDHB loss could trigger elevated IGF1R expression in the SDHB-intact tumors.

Approximately 20% of the wild-type GISTs in the current series did not exhibit elevated IGF1R expression. In the absence of augmented IGF1R expression, IGF-signaling pathways could potentially still be activated by elevated expression of other components of the IGF1-signaling pathway. For example, relative IGF2 expression was elevated among the IGF1R^low^ wild-type GISTs compared with the IGF1R^high^ wild-type GISTs. Inasmuch as HIF1A is a transcriptional activator of IGF2 [45], a positive correlation between HIF1A and IGF2 expression might have been predicted. However, HIF1A expression was approximately two- to threefold lower in the IGF1R^low^ population compared with the IGF1R^high^, the reciprocal of the IGF2 expression pattern in these populations.

The differential expression of IGF1R, as well as KIT, CD34, and INSR among the wild-type GIST groups, may reflect origins in distinct ICC populations. Detailed analyses of ICC progenitors in the mouse have identified KIT^low^CD34^+^INSR^+^IGF1R^+^ stem cells, KIT^high^CD34^+^INSR^+^IGF1R^+^ committed progenitors, and mature ICCs with a KIT^high^CD34^−^INSR^−^IGF1R^−^ phenotype [31,32]. Although the patterns of KIT, CD34, INSR, and IGF1R expression observed in this study do not match the murine ICC profiles, it is possible that the expression patterns of these markers differ between mouse and human, so that they may define discrete cell populations of origin of GISTs in humans. In addition, the KIT-mutant CD34^high^INSR^low^ IGF1R^low^ GISTs shared gene expression group 3 with the wild-type CD34^high^ INSR^low^ IGF1R^low^ GISTs, suggestive of possible commonalities of origin among these tumors.

Collectively, the molecular subgroups of wild-type GISTs defined in this study are of interest, as they may exhibit distinct clinical courses with differing sensitivities to targeted therapies. In comparison with KIT-mutant GISTs, the objective response to the kinase inhibitor imatinib is significantly lower among wild-type tumors (44–45% vs. 70–71%) [4–6]. Of note, SDH-deficient, IGF1R^high^ gastric GISTs show essentially no response to treatment with imatinib, but can respond to sunitinib [8,9]. In addition, IGF1R-targeted therapy of wild-type GIST is under investigation in clinical trials (e.g., NCT01560260), and the heterogeneity of IGF1R and IGF2 expression in the GIST groups may portend differential responses. Finally, heterogeneity among cells of origin may affect therapeutic responses, as has been suggested by murine GIST models [31]. Thus, the wild-type GIST molecular subtypes described herein may represent distinct clinical entities, so that their definition provides the foundation for further studies that could provide insights into improved tailored therapy and clinical management of GIST.

## Supporting Information

Table S1. Specimen numbers stratified by gene expression cluster, KIT/PDGFRA genotype, and tumor location.

Table S2. *P*-values associated with gene expression levels, stratified by cluster group and KIT/PDGFRA genotype.
